# Vole Foraging-Inspired Dynamic Path Planning of Wheeled Humanoid Robots Under Workshop Slippery Road Conditions

**DOI:** 10.3390/biomimetics10050277

**Published:** 2025-04-29

**Authors:** Hu Li, Yan Wang, Yixuan Guo, Jiawang Duan

**Affiliations:** 1 Guangdong Intelligent Robotics Institute, Dongguan 523808, China; 2 School of Mechanical Science and Engineering, Huazhong University of Science and Technology, Wuhan 430074, China; wangyn@hust.edu.cn (Y.W.);; 3 The Guangdong Provincial Key Laboratory of Human-Augmentation and Rehabilitation Robotics in Universities, Southern University of Science and Technology, Shenzhen 518055, China; guoyx@sustech.edu.cn

**Keywords:** path planning, humanoid robots, dynamic planning

## Abstract

A vole foraging-inspired dynamic path-planning method considering slippery road conditions is proposed for wheeled humanoid robots. Glazed and oily roads create a high risk of slipping for wheeled humanoid robots and hinder the realization of high-speed movement. But in a dynamic environment, road conditions such as material, texture, and attachments vary uncertainly in both space and time, and cannot be processed as quickly and easily as moving obstacles. Inspired by the process of voles searching for food, to address this challenge, a slip-risk-assessment method based on time–space decoupling is designed and integrated into a grid-based environmental model. On this basis, the dynamic path-planning model is constructed by combining the cost functions and constraints based on the slip-risk information. A two-level non-periodic cyclical dynamic planning mechanism is proposed based on conditional triggering. It adaptively and cyclically calls the global planning algorithm and the local re-planning algorithm according to the characteristics of environmental changes to autonomously avoid high-slip-risk areas and moving obstacles in real time. The experimental results show the effectiveness and practicality of the proposed planning method.

## 1. Introduction

Due to its operational flexibility and ability to adapt to complex working environments, the wheeled humanoid robot (WHR) has shown great potential and application prospects in industrial environments such as workshops. Generally, WHR consists of a wheeled mobile chassis mounted at the end of a single leg or two legs, along with the upper body of the conventional humanoid robot. As a special form of mobile robot, WHR integrates human-like functional features, such as multi-jointed arms for dexterous manipulation, visual and tactile sensors for perception, and the ability to communicate with humans through voice and gestures, enabling it to perform complex tasks in industrial settings. In applications, the effective path planning is a prerequisite for the autonomous execution of repetitive tasks. Mobile robots including WHRs need to use path-planning algorithms to determine the optimal path from the starting point to the target point in a given workshop environment [1,2]. However, path planning often faces many challenges and difficulties. First of all, the first condition for path planning is to obtain accurate environmental information [3]. Changes in the operating scene or the loss of part of the environment may cause the planned path to fail [4]. Secondly, dynamically moving obstacles will appear on the planned path and bring collision problems [5,6]. In addition, multiple basic requirements may need to be met at the same time, such as the shortest path, the highest safety, the lowest energy consumption, etc. [7].

In response to the above problems, many scholars have proposed various path-planning algorithms and related improvement methods. Path planning is usually divided into two stages: global planning and local planning [8,9]. Global planning aims to plan a global path from the starting point of the task to the target point in the entire working environment range [10]. On this basis, when the local environment changes, such as moving obstacles appearing in the local environment during operation, local planning adjusts the running path in real time to avoid obstacles, thereby ensuring work safety [11]. At present, common global planning algorithms include the heuristic algorithm [12,13], rapidly exploring random tree algorithm [14,15], reinforcement learning algorithm [16], etc. Local path-planning algorithms include the dynamic window method [17], artificial potential field method [18], etc. Each type of algorithm has its own characteristics. It is necessary to select a suitable path-planning algorithm according to the actual application scenario. In addition, many AI-driven methods, including deep learning and reinforcement learning, have been applied to directly solve the trajectory planning problem [19,20]. However, challenges, such as high computational cost, long training times, and a lack of robustness in real-world testing, remain limiting their application to practical real-world settings [21,22].

However, most path-planning algorithms of mobile robots only refer to two factors: static obstacles and moving obstacles in the operating environment [23]. For the WHR, road conditions will also affect the effectiveness of the planned path. The high center of gravity characteristics of WHR make them more sensitive to terrain and ground conditions and more likely to tip over. In response to the slipping problem caused by inclined terrain, a robust path-planning algorithm was proposed in [24] for a lunar rover traveling on a sand slope. In indoor working scenes, although the terrain is basically flat, factors such as path geometry, road material and texture, and road attachments will also affect the motion state of the WHR. Curves with smaller turning radii mean that the WHR needs a more lateral grip to avoid slipping or tipping over. To end this, the work in [25] proposed a dynamics-based anti-slip algorithm for the turning-trajectory planning of a differential wheeled mobile robot. When there is oil, mud or local water accumulation on the road, the wheels of the WHR will inevitably appear slipping when passing by. The current main solution is to improve the control method [26], weaken the dynamic disturbance caused by slippage, and enhance the robot’s robustness and motion stability. However, this type of post-compensation method has obvious limitations in its anti-skid ability. How to ensure that the planned path can adaptively change as the environment changes and achieve the effect of avoiding high-risk slip areas has become an important issue that needs to be solved urgently.

Inspired by the process of voles searching for food and returning to nests, this paper proposes a vole foraging-inspired dynamic path-planning method for a WHR working in a workshop with slippery road conditions and moving obstacles. When voles are foraging, they will use their keen vision, hearing, and sense of smell to perceive the surrounding environment and identify potential dangers, such as the smell of predators, abnormal sounds, or moving objects. Based on this, they not only dynamically adjust the local path during the journey to avoid predators in real time, but also adjust the global path from the nest to the foraging point from time to time to avoid risks in advance. Inspired by this, a two-level non-periodic cyclical dynamic planning mechanism is constructed to assist the WHR in dealing with workshop slippery road conditions. The main contributions are summarized as follows.

(1) A slip-risk-assessment method based on time–space decoupling is designed to improve the grid-based environment model. Thanks to this, compared with traditional path-planning methods such as [7,12], the impact of road conditions such as material, texture, and road attachments is also considered, thereby enhancing the effectiveness and practicality of the planned path.

(2) By introducing the time-varying slip-risk factors into the cost functions and constraints of global planning and local re-planning, a novel dynamic path-planning model is constructed. Unlike the anti-slip strategy based on post compensation such as [26], the proposed method can ensure that WHR can effectively avoid high-risk slip areas in dynamic environments.

(3) A two-level non-periodic cyclical dynamic planning mechanism is proposed for scenes where road conditions and moving obstacles change uncertainly at different time scales. With the help of the designed trigger condition, its planning will be more efficient and accurate than traditional global–local coupling path-planning mechanisms [8,9].

## 2. Problem Formulation

### 2.1. Grid-Based Environment Modeling

The operating environment model needs to be established before planning paths. Common environmental modeling methods include the visibility graph method, Voronoi diagram method, grid method, free space method, etc. Among them, the grid method has a simple structure, is easy to program and implement, and can easily support the update and change of dynamic environment. Specifically, the map is first constructed after using sensors such as lidars and cameras to identify obstacles, roads, boundaries, and other environmental information. Then, the constructed map is gridded, where the grid size is set according to the size of the mobile robot. Finally, the following coordinate system is constructed to mark the location of each grid and its traffic status.

(1) $$\Theta =\left[\begin{array}{cccc} \theta_{1,1} & \theta_{1,2} & \cdots & \theta_{1,n}\\ \theta_{2,1} & \theta_{2,2} & \cdots & \theta_{2,n}\\ \vdots & \vdots & \ddots & \vdots \\ \theta_{m,1} & \theta_{m,2} & \cdots & \theta_{m,n} \end{array}\right]$$

where $\theta_{i,j}=\left\{\left(x_{i},y_{j}\right),A_{i,j}\left(t\right)\right\}$ with ∀i=1,2,⋯,m and ∀j=1,2,⋯,n, $x_{i}$ and $y_{j}$ denote the horizontal and vertical coordinates of the center point of the grid, and $A_{i,j}\left(t\right)$ denotes the traffic status of the grid where $\left(x_{i},y_{j}\right)$ is located at time *t*.

**Figure 1:**
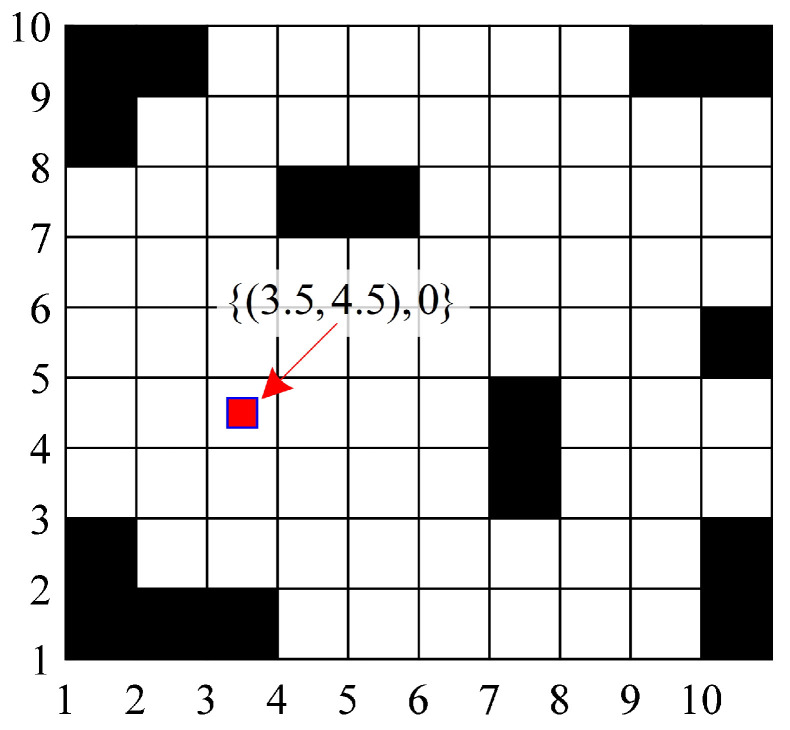
An example of a grid environment model.

An example of a grid environment model is shown in Figure 1. The black grids represent fixed obstacles, where the traffic status is prohibited access, i.e., $A_{i,j}\left(t\right)=\infty$. The white grid represents the area where the mobile robot is allowed to pass, where the traffic status here is marked as $A_{i,j}\left(t\right)=0$.

### 2.2. Problem Formulation

The considered working environment is a semi-structured scene with clear environmental characteristics, a clear spatial layout, but uncertain road attachments, such as production workshops, warehouses, etc. On the road, oil or water will inevitably appear and affect the passage of the mobile robot. These road attachments are unpredictable but can be observed by sensors. In addition, staff and other mobile devices are allowed to move within the lane of the WHR at any time, and they are regarded as mobile obstacles during path planning.

In this letter, the grid method is employed to model the environment for the considered dynamic semi-structured scene. Here, the same location (i,j) has a different traffic status at a different time *t*. It is able to switch between two states: prohibited $A_{i,j}\left(t\right)=\infty$ and allowed $A_{i,j}\left(t\right)=0$, in most existing trajectory-planning algorithms. In fact, there are also restricted traffic conditions, $0<A_{i,j}\left(t\right)<\infty$. For example, roads with water or oil pollution allow WHR to pass, but may cause slippage or even instability.

For voles, they also face situations similar to the above-mentioned scenarios during the foraging process. On the path from their burrows to the foraging locations, they may encounter puddles or piles of stones, which can affect their normal movement. In addition, other organisms or natural enemies may also appear on the round-trip path at any time, preventing voles from foraging and walking normally. Therefore, during the foraging process, voles will make full use of their eyes, noses, and ears to obtain information about the surrounding environment. Then, they will continuously adjust the global and local foraging paths based on their own thinking to ensure their own safety and completion of the foraging task.

Inspired by the above-mentioned behavior of voles during the foraging process, the objective of this paper is to design a dynamic path-planning method such that:

(1) The WHR can successfully reach the target position from the starting position with the shortest path length;

(2) The WHR will not collide with fixed obstacles or moving people or objects during operation;

(3) The WHR has the lowest risk of slippage.

## 3. Slip Risk Assessment

The anti-slip performance of the WHR can be characterized by the friction coefficient μ between the tire and the road surface, as shown below.

(2) $$\mu =F_{\mu }/F_{N}$$

where $F_{\mu }$ represents the friction force experienced by the tire and $F_{N}$ represents the normal load experienced by the tire.

The larger the friction coefficient is, the greater the friction force will be under the given load in the vertical direction of the tire. Then, the more difficult it is for the tire to slide relative to the road surface.

Employing the friction coefficient μ, a slip-risk-assessment criterion is designed, as follows.

(3) $$s=f\left(\mu \right)=\left\{\begin{array}{cc} \infty , & 0\leq \mu \leq \mu_{0}\\ e^{k/\mu }, & \mu >\mu_{0} \end{array}\right.$$

where *s* represents the slip risk, μ represents the comprehensive friction coefficient, $\mu_{0}$ represents the critical value of μ, and *k* represents the correction coefficient. When $0\leq \mu \leq \mu_{0}$, due to the small value of μ, WHR is prone to slip. Therefore, a relatively large value is assigned to *s*. In the second case, the value of *s* is obtained in the form of an exponent.

For the considered working environment, the road surface material and texture are unchanged in the time dimension, but change in the space dimension. Road attachments including water stains and oil stains change dynamically in both the time dimension and the space dimension. Therefore, the slip risk faced by a given WHR changes uncertainly and is difficult to quantify in advance.

A slip-risk-assessment algorithm based on time–space decoupling is proposed. It consists of three links: offline calibration, online pre-adjustment, and feedback correction, as detailed below.

*Step 1: Offline calibration.*

Before planning the path, a grid map with a slip-risk coefficient is established, that is, A(t)=s for any grid without fixed obstacles. Here, only the influence of the road-surface material and texture at each grid is measured, assuming that there is no road attachment. The slip-risk coefficients imposed by various possible road attachments are summarized into a database Ψ. For this, the friction coefficient μ of the areas except for those with fixed obstacles required to evaluate the slip risk is measured offline using a professional friction-coefficient test instrument.

*Step 2: Online pre-adjustment.*

The image recognition system periodically extracts and analyzes the road attachments in the working environment at a time interval $\Delta T_{A}$. Then, the slip-risk coefficient at each grid point in the grid map is corrected based on the characteristic information of the road attachments and the database Ψ.

*Step 3: Feedback correction.*

The slip risk at each grid point on the driving path is recalibrated based on the longitudinal slip rate λ and lateral slip amount α of the WHR. The feedback correction rules are as follows:

(4) $$s=\left\{\begin{array}{cc} \infty , & \mathrm{if}\;\left|\lambda \right|\leq \lambda_{\max}\;\mathrm{or}\;\left|\alpha \right|\leq \alpha_{\max}\\ f(\mu ), & \mathrm{else} \end{array}\right.$$

where $\lambda_{\max}$ and $\alpha_{\max}$ are the thresholds for λ and α, respectively.

The longitudinal slip rate λ can be characterized by the longitudinal velocity loss, as shown in the following formula.

(5) $$\lambda =\left(\omega r-v\right)/v$$

where ω represents the angular velocity of the driving wheel, *r* represents the radius of the driving wheel, and *v* represents the actual operating speed of the mobile robot. Specifically, ωr−v represents the speed deviation of the wheel. By dividing by *v*, we can obtain the slip rate relative to the actual speed.

The lateral slip amount α is calculated as

(6) α=arctan(ζ/v)

where ζ represents the lateral speed of the mobile robot due to lateral slip, and *v* represents the theoretical operating speed of the WHR. This formula follows the same logic as (5). However, since the lateral slip is taken into consideration, an additional arctangent calculation is added.

## 4. Vole Foraging-Inspired Dynamic Path Planning

### 4.1. Dynamic Path-Planning Model

In this section, the global path-planning model is firstly designed to ensure that the WHR can move to the task target point efficiently, smoothly, and safely. Next, the local re-planning model is introduced to achieve dynamic obstacle avoidance. In particular, the above two plans are based on grid environment models with slip-risk information.

*(1) Global path-planning model*

The global path planning is formulated as a multi-objective optimization problem with the dynamic constraint as follows:

(7) $$\begin{array}{c} \min J_{G}\left(\Delta T_{A}\right)=\left(J_{1}\left(\Delta T_{A}\right),J_{2}\left(\Delta T_{A}\right),J_{3}\left(\Delta T_{A}\right),J_{4}\left(\Delta T_{A}\right)\right)\\ s.t.\;P\cap R_{I}\left(\Delta T_{A}\right)=\emptyset \end{array}$$

where $J_{i}\left(\Delta T_{A}\right)$(i=1,2,3,4) represents the cost function related to path length, the path smoothness, the collision risk, and the slip risk, and $P\cap R_{I}\left(\Delta T_{A}\right)=\emptyset$ represents the constraint that the planned path does not intersect with any inaccessible area with obstacles, which is used to ensure that the WHR completes the task under the premise of safety, where $R_{I}\left(\Delta T_{A}\right)$ represents the inaccessible area with obstacles, and $\Delta T_{A}$ is the road attachment detection time window.

To ensure that the global path planning can adapt to the dynamically changing WHR operating environment, the environmental constraint modeling needs to be updated under the constraints of the road-attachment detection time window $\Delta T_{A}$, that is, after marking the slip occurrence area, it is defined as an inaccessible area $R_{I}\left(\Delta T_{A}\right)$. Its specific definition expression is as follows:

(8) $$\begin{array}{c} R_{I}\left(\Delta T_{A}\right)=r_{I}\cup {ℜ}_{I}\left(\Delta T_{A}\right)\\ s.t.\left\{\begin{array}{c} {ℜ}_{I}\left(\Delta T_{A}\right)=\sum ℜ\\ ℜ=f\left(\Delta T_{A}\right)-f_{\min}\leq 0 \end{array}\right. \end{array}$$

where $r_{I}$ represents the grid area where the fixed obstacle is located, ${ℜ}_{I}\left(\Delta T_{A}\right)$ represents the area composed of grids that meet the conditions *ℜ* within the road-attachment detection time window $\Delta T_{A}$, $f(\Delta T_{A})$ represents the slip-risk coefficient within $\Delta T_{A}$, and $f_{\min}$ represents the allowable slip during the operation of the WHR.

The path length $J_{1}$ is calculated from the Euclidean distance of the line segments that make up the path. Assume that there are *n* nodes on the planned path, so there are n−1 polylines. The length of the path is the sum of the lengths of all polylines between the starting point and the target point. Its specific definition is

(9) $$J_{1}=\sum_{i=1}^{n-1}\sqrt{{\left(x_{i+1}-x_{i}\right)}^{2}+{\left(y_{i+1}-y_{i}\right)}^{2}}$$

where $\left(x_{i},y_{i}\right)$ represents the position coordinate of a node in the path.

The path smoothness $J_{2}$ refers to the deflection angle of the planned path at the bend, whose definition is:

(10) $$J_{2}=\frac{1}{n-2}\sum_{i=2}^{n-1}\exp(-\alpha_{i}^{i+1})$$

where $\alpha_{i}^{i+1}$ represents the angle between two adjacent polyline segments, and its value is obtained by calculating the inverse function of $\cos(\alpha_{i}^{i+1})$.

(11) $$\cos\left(\alpha_{i}^{i+1}\right)=\frac{d_{i-1}^{i}\left(x\right)d_{i}^{i+1}\left(x\right)+d_{i-1}^{i}\left(y\right)d_{i}^{i+1}\left(y\right)}{\sqrt{d_{i-1}^{i}{\left(x\right)}^{2}+d_{i-1}^{i}{\left(y\right)}^{2}}\sqrt{d_{i}^{i+1}{\left(x\right)}^{2}+d_{i}^{i+1}{\left(y\right)}^{2}}}$$

with

(12) $$\left\{\begin{array}{c} d_{i-1}^{i}\left(x\right)=x_{i-1}-x_{i}\\ d_{i-1}^{i}\left(y\right)=y_{i-1}-y_{i}\\ d_{i}^{i+1}\left(x\right)=x_{i+1}-x_{i}\\ d_{i}^{i+1}\left(y\right)=y_{i+1}-y_{i} \end{array}\right.$$

where $\left(x_{i-1},y_{i-1}\right)$, $\left(x_{i},y_{i}\right)$, and $\left(x_{i+1},y_{i+1}\right)$ are the position coordinates of three adjacent nodes in the path.

The collision risk $J_{3}$ is evaluated by calculating the distance between the WHR and obstacles, as follows

(13) $$J_{3}=\left\{\begin{array}{cc} 0, & l_{i}\geq L_{\min}\\ \sum_{i=1}^{z-1}e^{-l_{i}/L_{\min}}, & \mathrm{otherwise} \end{array}\right.$$

(14) $$l_{i}=\min\left\{\sqrt{{\left(x_{j}-x_{k}\right)}^{2}+{\left(y_{j}-y_{k}\right)}^{2}}\left|k=1,2\right.,\ldots ,\Pi \right\}$$

where $l_{i}$ represents the minimum distance between the path polyline segment and the obstacle, $\left(x_{i},y_{i}\right)$ represents the coordinates of a certain point in the polyline segment, $\left(x_{k},y_{k}\right)$ represents the center coordinates of an obstacle in the grid environment model, Π represents the number of obstacles, $L_{\min}$ represents the ultimate safe distance between WHRs and obstacles, and *z* represents the number of nodes in the path.

The slip risk $J_{4}$ is designed to quantify the probability of slip when the WHR runs on the planned path. The specific definition of path slip risk is

(15) $$J_{4}=\sum_{i=2}^{n-1}s_{i}$$

where $s_{i}$ represents the slip-risk coefficient of the node on the path planned by the WHR.

(2) Local re-planning model

Local path re-planning in the presence of moving obstacles can be formulated as

(16) $$\begin{array}{c} \min J_{\Xi }\left(\Delta t_{a}\right)=\left(J_{5}\left(\Delta t_{a}\right),J_{6}\left(\Delta t_{a}\right)\right)\\ s.t.\;J_{\Xi }\left(\Delta t_{a}\right)\propto TCR_{car}\left(\Delta t_{a}\right) \end{array}$$

where $J_{5}\left(\Delta t_{a}\right)$ represents the collision-avoidance cost function, used to ensure that the re-planned path allows the robot to successfully avoid moving obstacles, $J_{6}\left(\Delta t_{a}\right)$ represents the deviation-path cost function, used to guide the WHR to return to the previously planned global path as soon as possible after avoiding the mobile obstacle, and $TCR_{car}\left(\Delta t_{a}\right)$ represents a collision-risk-assessment model used to evaluate the degree of threat posed by passing robots or other moving obstacles near a WHR. $J_{\Xi }\left(\Delta t_{a}\right)\propto TCR_{car}\left(\Delta t_{a}\right)$ represents a path re-planning model established using the decision-making basis provided by the collision-risk-assessment model, where $\Delta t_{a}$ indicates the current collision detection time window. The size of $\Delta t_{a}$ depends on many factors, including the response speed of the WHR hardware, the sensing range of the onboard sensing equipment, and the mobility of the WHR.

The collision-avoidance cost function $J_{5}\left(\Delta t_{a}\right)$ is established as shown in the following formula, which is mainly composed of the obstacle distance function and the dynamic obstacle-avoidance function:

(17) $$J_{5}\left(\Delta t_{a}\right)=d\left(\Delta t_{a}\right)+v_{a}\left(\Delta t_{a}\right)$$

where $d(\Delta t_{a})$ represents an evaluation index related to the calculated distance between the current WHR and the obstacle under the constraints of the collision detection time window, and $v_{a}\left(\Delta t_{a}\right)$ represents an evaluation index related to the azimuth between the WHR and the obstacle.

The deviation-path cost function $J_{6}\left(\Delta t_{a}\right)$ is established, which is mainly composed of the WHR’s orientation function. It is composed of the evaluation function of current speed and size. The specific expression is as follows:

(18) $$J_{6}\left(\Delta t_{a}\right)=h\left(\Delta t_{a}\right)+v\left(\Delta t_{a}\right)$$

where $h(\Delta t_{a})$ represents an evaluation index related to the azimuth between the global path and the local path, and $v(\Delta t_{a})$ represents an evaluation index related to the velocity of the WHR.

### 4.2. Two-Level Non-Periodic Cyclical Dynamic Planning

The global planning and local re-planning of the robot’s motion trajectory are executed cyclically to cope with the dynamic changes in the operating environment. It is worth emphasizing that the appearance and disappearance of road attachments and mobile obstacles are not fixed periods, and have different time scales. In order to ensure planning efficiency, a new two-level non-periodic cyclic dynamic planning method based on conditional triggering is designed, as shown in Algorithm 1. Specifically, the path planning of the WHR considering slip can be divided into five steps, which will be described in detail below.
  **Algorithm** **1:** Two-level non-periodic cyclical dynamic planning         **Input**:grid map of the experimental scene, starting point $\left(x_{1},y_{1}\right)$, target point $\left(x_{n},y_{n}\right)$, initial slip-risk coefficient of each grid point **1**   **Main loop** **2**      **While** the WHR has not reached the target point **3**          Detect the operating environment and the state of the WHR; **4**          Refresh the operating environment model by (1)–(6); **5**          **If** the trigger condition is met **6**              Plan the global path by (7)–(15); **7**              Re-plan the local path by (16)–(18); **8**          **Else** **9**              Re-plan the local path by (16)–(18);**10**          **End if****11**      **End while****12**  **End loop**

*Step 1: Detect the operating environment and the state of the WHR.*

Through detection equipment such as automatic identification systems, vision systems, lidars, or airborne cameras, obtain basic information about the WHR’s operating environment, such as fixed-obstacle position information and road-surface roughness information, etc., and obtain real-time information about moving obstacles.

*Step 2: Refresh the operating environment model.*

Based on the observed information, the grid method in Section 2 and the slip-risk-assessment method in Section 3 are combined to update the environmental model.

*Step 3: Determine whether the trigger condition is met.*

The trigger conditions are designed as follows:

(1) No global path exists;

(2) The road attachment at any grid $\left(x_{i},y_{j}\right)$ in the operating environment changes and the robot stops moving, i.e., for ∀i=1,2,⋯,m and ∀j=1,2,⋯,n, there is

(19) $$\left(A_{i,j}\left(t_{now}\right)\neq A_{i,j}\left(t_{now}-\Delta T_{A}\right)\right)\&\left(t_{now}=T_{end}\right)$$

where *m* and *n* denotes the upper limitation of the following coordinate system of grid environment model, $t_{now}$ denotes the current time, $\Delta T_{A}$ denotes road-attachment detection time window, and $T_{end}$ denotes the robot operation suspension time.

If either of the above two trigger conditions is met, the global path-planning task is executed; otherwise, the local path-planning task is executed.

*Step 4: Plan the global path.*

The global trajectory-planning model (7) is solved using an optimization algorithm to obtain the optimal path in the current environment. The optimization algorithms that can be used include ant colony optimization, particle swarm optimization, differential evolution algorithm, etc.

*Step 5: Re-plan the local path.*

By evaluating the collision-risk coefficient of moving obstacles within the perception range of the running WHR, determine whether to perform dynamic avoidance and the order of avoidance. When the collision risk exceeds the threshold, the local re-planning model is solved to fine-tune the planned global path to ensure the safety of the WHR. The dynamic window optimization algorithm can be used as the solver of the local path re-planning model (16).

**Remark** **1.** *For the method of stopping and waiting to deal with dynamic obstacles, due to the different motion states of the dynamic obstacles, it is relatively complicated to calculate the stopping acceleration and the stopping position. Moreover, it may be difficult to handle complex situations. On the contrary, the proposed method can actively carry out path planning to complete obstacle avoidance, which can improve the movement efficiency and has a wider adaptability.*

**Remark** **2.** *For the proposed method, the important improvement of path-planning process is reflected in two aspects. One is the improvement of the planning problem model by incorporating the changes in road-slip conditions, and the other is the design of a non-periodic calling mechanism for global planning and local planning to facilitate the planning of a safe path without slipping. Here, the existing excellent algorithms can be directly introduced to solve global planning problems or local planning problems. Therefore, the proposed method can further improve the safety of WHR traveling in workshops with slippery roads on the basis of inheriting the advantages of existing planning algorithms.*

## 5. Results and Discussion

### 5.1. Results

**Figure 2:**
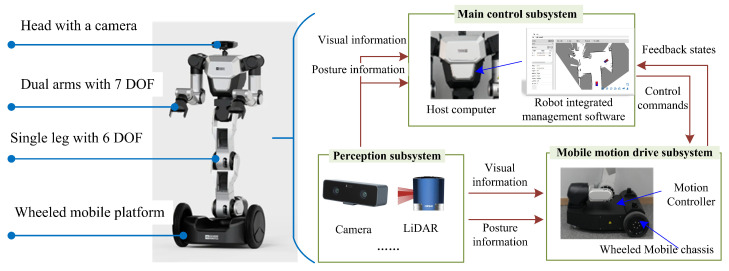
The experimental platform.

The WHR considered in the simulation experiment is shown in Figure 2, with reference to the RB-Y1 robot of RAINBOW ROBOTICS. The considered WHR consists of dual arms with seven degrees of freedom, a single stand with six degrees of freedom, a wheeled mobile platform, and a head with a camera. Its modules related to trajectory planning mainly include a perception subsystem, mobile motion-drive subsystem, and main control subsystem.

(1) The perception subsystem consists of modules such as binocular cameras, laser radars, and digital signal processors, which are used to obtain the surrounding environment information and the state information of WHR.

(2) The motion-drive system consists of a mobile chassis with wheels, a motion controller, etc. According to the signal received from the obstacle-avoidance system, a mobile motion controller is used to control the position and speed of the WHR. The dimensions of the mobile chassis are 662.62 mm long and 580 mm wide.

(3) The main control subsystem consists of a host computer with robot integrated management software, in which the path-planning algorithm is deployed. The main control subsystem generates motion instructions based on the environmental information and robot state information obtained by the perception subsystem using the proposed path-planning algorithm and sends them to the motion-drive subsystem.

**Figure 3:**
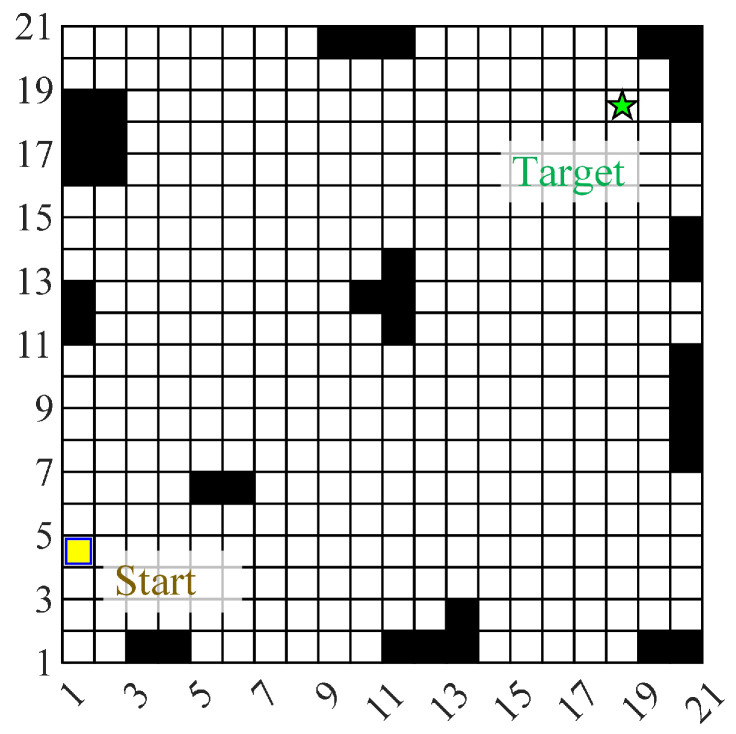
A grid map of the experimental scene.

**Figure 4:**
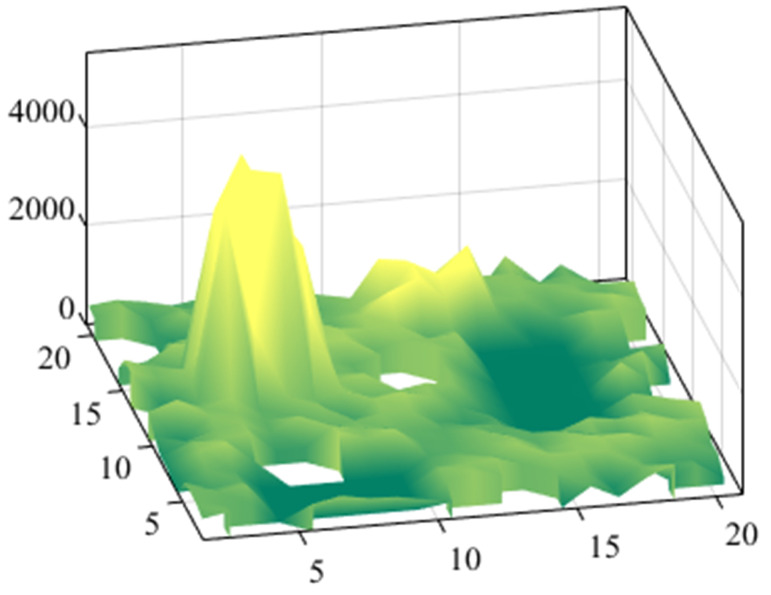
The slip risk of the experimental scene.

The experimental scenario with a total size of 14 m × 14 m. The operation scene consists of boundary walls, fixed obstacles, mobile obstacles, and roads. After the experimental test begins, the WHR needs to move from the starting point to the target point and return along the original route. Figure 3 shows the grid map of the experimental scene, where the black area represents static obstacles, the white area is the potential passable area, the yellow square represents the starting point, and the green five-pointed star represents the target point. The size of the grid is 70 cm × 70 cm. The initial slip-risk coefficient of each grid point is shown in the Figure 4. During the reciprocating operation, before the WHR performs the ninth movement operation, oil will appear in the working area. When WHR detects the above changes, it will send a reminder message to the responsible personnel, and then the oil stains will be manually removed after a period of time. Particle swarm optimization and dynamic window optimization are employed as solvers for global optimization and local re-planning of trajectories, respectively.

**Figure 5:**
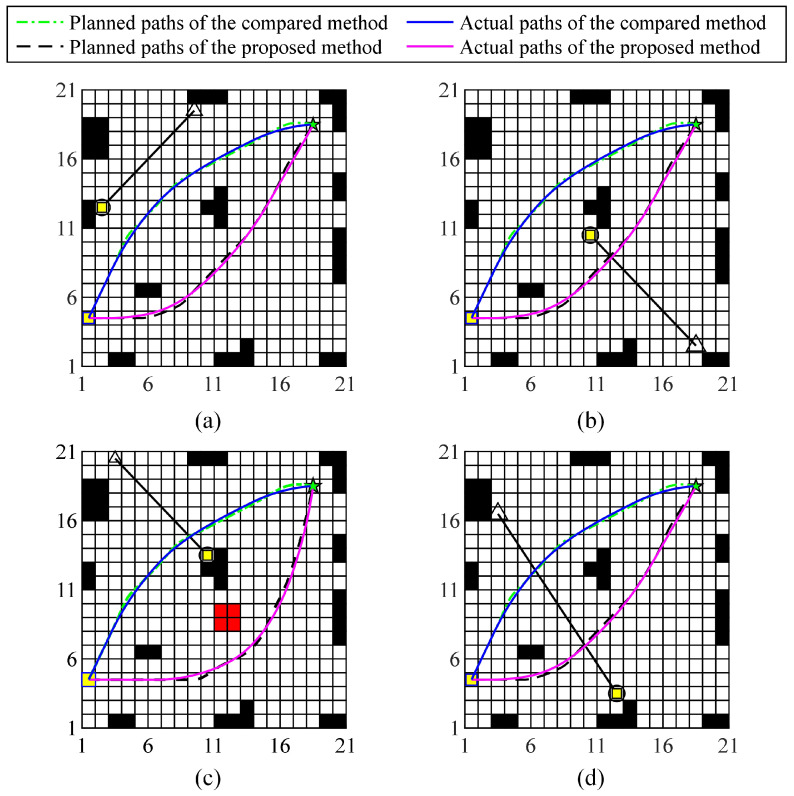
The paths of WHR during several key time periods. (**a**) The first movement operation. (**b**) The seventh movement operation. (**c**) The ninth movement operation. (**d**) The fifteenth movement operation.

**Figure 6:**
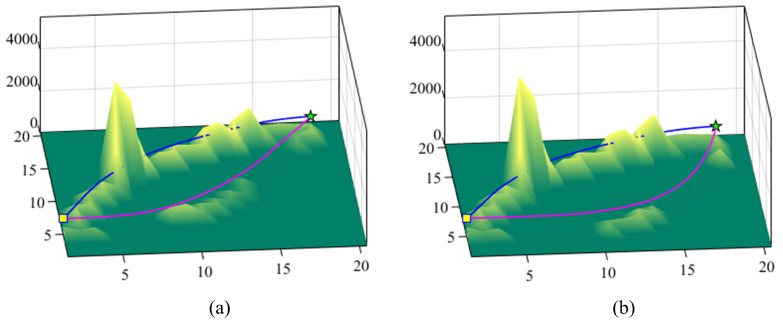
The slip state of WHR during several key time periods. (**a**) The seventh movement operation. (**b**) The ninth movement operation (the blue line represents the trajectory planned by the comparison algorithm, and the red line represents the trajectory planned by the proposed algorithm).

The paths of WHR at several key periods are shown in Figure 5. At the same time, Figure 6 shows the slip state of WHR in several key periods.

### 5.2. Discussion

**Figure 7:**
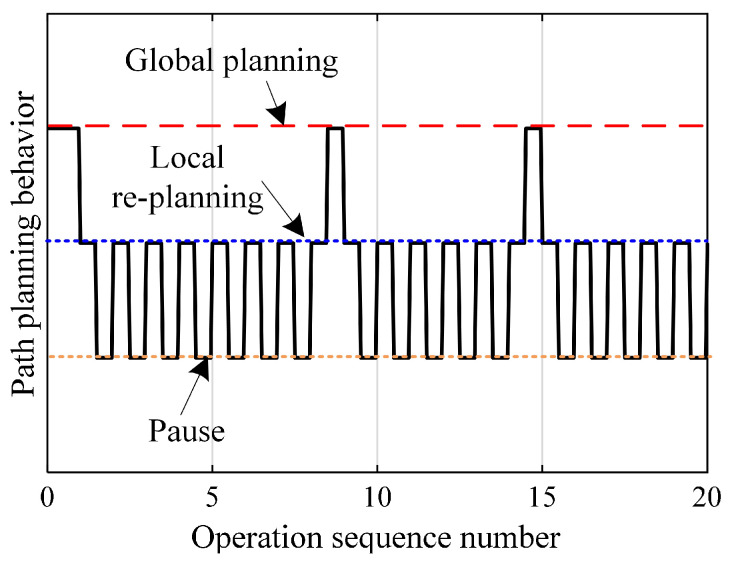
The dynamic switching process of the path-planning behavior.

As can be seen from Figure 5, the WHR path planned by the comparison planning algorithm remains unchanged in each moving operation. But, the motion path generated by the proposed planning method is not only different from the route generated by the comparison algorithm, but also changes between different moving operation cycles. This means that the proposed planning method can dynamically adjust the motion path according to the changes in road conditions. As shown in Figure 5c, when performing the ninth moving operation, an oily area represented by the red area appears. Fortunately, the path generated by the proposed method can avoid this high-risk area for slip. The slip state of WHR during the seventh and ninth moving operations can be found in Figure 6a and Figure 6b, respectively. The blue line and the red line represent the trajectory planned by the comparison algorithm and the trajectory planned by the proposed algorithm, respectively. It can be seen that the slip risk of WHR on the trajectory planned by the comparison algorithm is significantly higher than that on the trajectory planned by the proposed algorithm. The planned path considering the road slip risk can effectively and autonomously avoid the high-slip-risk area. Therefore, the overall slip risk on the path is at a low level. In addition, when a change in road conditions is detected, the proposed planning method re-plans the global path to achieve minimum slip. Due to the continuous refreshing of the road-attachment detection time window, the planning system of WHR continuously switches between global planning and local planning, as shown in Figure 5, and the dynamic switching process between global planning and local planning is shown in Figure 7. These results show that the proposed method can ensure that WHR achieves minimum slip.

## 6. Conclusions

In order for the WHR to achieve collision-free and minimal slip motion in environments with slippery roads and moving obstacles, a vole foraging-inspired dynamic path-planning method is proposed. Specifically, we propose the slip-risk-assessment method by introducing the environmental information and the state information of the robot based on time–space decoupling. Based on the above technique, the grid-based environment model with the slip-risk coefficient was first constructed, which incorporates the impact of road conditions such as material, texture, and road attachments. Then, global planning and local re-planning problems of the path are modeled based on the cost functions and constraints considering the slip-risk information. The global–local coupling path-planning problem was solved by the designed two-level non-periodic cyclical dynamic planning mechanism, which guarantees that the robot can actively switch between global planning and local re-planning according to the obtained information. The experiments show that based on the proposed algorithm, the robot can autonomously select the shortest and smoothest path that avoids areas with high slip risks, while also avoiding obstacles in real time.

This paper focuses on how wheeled humanoid robots plan safe paths in workshop slippery road conditions. In this scenario, road conditions can be observed by measurement equipment other than robots. Future research scenarios can be expanded from workshops to outdoors, or this algorithm can be improved through deep learning.

## Data Availability

The data generated and/or analyzed during the current study are not publicly available for legal/ethical reasons but are available from the corresponding author upon reasonable request.

## Abbreviations

The following abbreviations are used in this manuscript:

WHR	Wheeled humanoid robots
